# Characterizing practice-dependent motor learning after a stroke

**DOI:** 10.1007/s10072-024-07815-y

**Published:** 2024-11-06

**Authors:** Annibale Antonioni, Nicola Cellini, Andrea Baroni, Giulia Fregna, Nicola Lamberti, Giacomo Koch, Fabio Manfredini, Sofia Straudi

**Affiliations:** 1 Department of Neuroscience and Rehabilitation, University of Ferrara, Via Ludovico Ariosto 35, Ferrara, Ferrara 44121 Italy; 2 Doctoral Program in Translational Neurosciences and Neurotechnologies, University of Ferrara, Ferrara, 44121 Italy; 3 Department of General Psychology, University of Padua, Padua, Italy; 4 Padova Neuroscience Center, University of Padua, Padua, Italy; 5 Department of Neuroscience, Ferrara University Hospital, Ferrara, 44124 Italy; 6 Center for Translational Neurophysiology of Speech and Communication (CTNSC), Italian Institute of Technology (IIT), Ferrara, 44121 Italy; 7 Non Invasive Brain Stimulation Unit, Istituto di Ricovero e Cura a Carattere Scientifico Santa Lucia, Rome, 00179 Italy

**Keywords:** Stroke, Motor learning, Inter-limb transfer, Finger tapping Task (FTT), Rehabilitation

## Abstract

**Background:**

After stroke, patients must learn to use residual motor function correctly. Consistently, motor learning is crucial in stroke motor recovery. We assessed motor performance, practice-dependent on-line motor learning, and factors potentially affecting them in stroke patients.

**Methods:**

This is a cross-sectional observational study. Twenty-six patients with first brain stroke leading to upper limb motor deficit in the subacute or chronic timeframe were enrolled. They performed a Finger Tapping Task (FTT) with both the affected and unaffected limbs. We assessed how patients learn to perform motor tasks despite the motor deficit and the differences in performance between the unaffected and affected limbs. Furthermore, by randomizing the order, we evaluated the possible inter-limb transfer of motor learning (i.e. transfer of a motor skill learned in one limb to the opposite one). Moreover, sleep, attention, anxiety, and depression were assessed through specific tests and questionnaires.

**Results:**

Improved FTT accuracy and completed sequences for the affected limb were observed, even if lower than for the unaffected one. Furthermore, when patients initially performed the FTT with the unaffected limb, they showed higher accuracy in subsequent task completion with the affected limb than subjects who started with the affected limb. Only anxiety and attentional abilities showed significant correlations with motor performance.

**Conclusions:**

This work provides relevant insights into motor learning in stroke. Practice-dependent on-line motor learning is preserved in stroke survivors, and an inter-limb transfer effect can be observed. Attentional abilities and anxiety can affect learning after stroke, even if the effect of other factors cannot be excluded.

## Introduction

Cerebral stroke is a leading cause of death and long-term disability worldwide [1]. Despite recent advances in its management, stroke patients with long-term outcomes are still very common [2]. Thus, identifying effective strategies to ensure complete functional recovery is crucial. Upper limb motor deficits are critical because of their impact on activities of daily living (ADL) and the resulting loss of independence. Indeed, restoring post-stroke upper limb motor patterns is challenging due to the complexity of motor behaviours [3]. Importantly, after stroke, the central nervous system (CNS) undergoes increased neuroplasticity aimed at promoting the neural reorganisation that accounts for spontaneous recovery (i.e. the return to the premorbid motor level). However, it manifests within a restricted time interval and, often, does not lead to full restoration of impaired function [4]. Indeed, stroke patients in the chronic phase are often characterized by persistent and even severe outcomes [5]. Since several weeks after stroke the neurobiological mechanisms associated with true recovery are over, it is fundamental to properly use residual effectors to accomplish tasks (i.e. compensation) [6]. Notably, recent advances in motor learning, i.e. processes aimed at learning and refining new skills through practice, could play a fundamental role in stroke rehabilitation [7]. Consistently, several studies proved that chronic stroke patients not only retain the ability to learn motor skills, e.g. a motor sequence with the affected arm, but also that this is crucial for maintaining their ADLs [8, 9]. Such simple tasks allow not only to assess stroke patients’ motor abilities, but also to study their improvement during the task itself (i.e. on-line learning), which is a form of practice-dependent learning relevant from a neurorehabilitation perspective [10]. Indeed, motor learning encompasses separate phases: acquisition, marked by performance enhancements during practice (on-line changes), and retention, evident in performance after a break (off-line changes) [11]. Recent studies confirmed that chronic stroke patients exhibit both acquisition and retention of motor skills. However, the results are often heterogeneous, reasonably due to a complex interplay between numerous factors [8, 9]. Consistently, although the neurophysiological mechanisms are partially unknown, numerous reports suggest a role of multiple factors on motor skills acquisition, e.g. psycho-emotional conditions (e.g. anxiety and depression, attentional skills) [12, 13], sleep [14], and smoking [15]. Importantly, stroke patients are often characterized by post-stroke depression, sleep disturbances, and cognitive impairment, which adversely affect their motor performance [13, 16–18]. Thus, collecting data on motor learning strategies after stroke, both in the subacute and in the chronic phase, and on factors that may influence these mechanisms is a fundamental goal of modern neurorehabilitation [5]. Indeed, considering their clinical stability, these stroke timeframes are the most suitable for assessing the characteristics of motor learning and the role of factors that could affect it.

Here, we evaluated motor performance and practice-dependent on-line motor learning in stroke patients with an upper limb motor deficit in a subacute and chronic timeframe. These periods are critical for optimizing compensation strategies, as post-stroke neuroplasticity facilitating true recovery is markedly reduced. Participants performed a simple motor task using unaffected and affected limbs. We assessed how patients learn to perform motor tasks despite the motor deficit and the differences in performance between the unaffected and affected limbs. Furthermore, by randomizing the order, we evaluated the possible inter-limb transfer of motor learning (i.e. transfer of a motor skill learnt in one limb to the opposite one), and investigated its characteristics between the two limbs [19]. We collected data on factors reasonably influencing motor learning, e.g. mood, attention, and sleep. The aim was to assess the characteristics of this complex neurophysiological mechanism after stroke and the influence of potentially modifiable factors.

## Materials and methods

### Study design

We performed a cross-sectional observational clinical study at the Ferrara University Hospital between December 2022 and April 2023. All the procedures were conducted according to the Declaration of Helsinki and approved by the Area Vasta Emilia-Centro Ethics Committee (Protocol ID 641/2018/Sper/AOUFe) [20]. This study is reported according to the STROBE guidelines.

### Subjects recruitment

Subjects admitted to the Ferrara University Hospital were recruited. Subjects’ eligibility was assessed based on inclusion and exclusion criteria. Subjects were interviewed and informed about the study’s objectives and procedures, and informed consent was obtained. Inclusion criteria were: males and females aged ≥ 18 years; diagnosis of first cerebral stroke verified by neuroimaging in the subacute (between one and six months from the onset) or chronic period (after at least six months from the onset); upper limb motor deficit, but not preventing the execution of computer tasks, as assessed by a score > 39 on the Action Research Arm test scale [21]; at least five years of education. Exclusion criteria were: cerebellar or bilateral cerebral stroke; any medical or neurological conditions, other than stroke, that could prevent the safe completion of the study protocol or receipt of informed consent (including aphasia and apraxia); visual or auditory disturbances that could hinder the task performance; diagnosis or previous history of substance abuse, traumatic brain injury, neurosurgical interventions, severe psychopathology, cognitive decline or dementia (as assessed by a Mini Mental State Examination score < 24) [22]; pregnancy.

### Procedure

#### Motor task

**Fig. 1:**
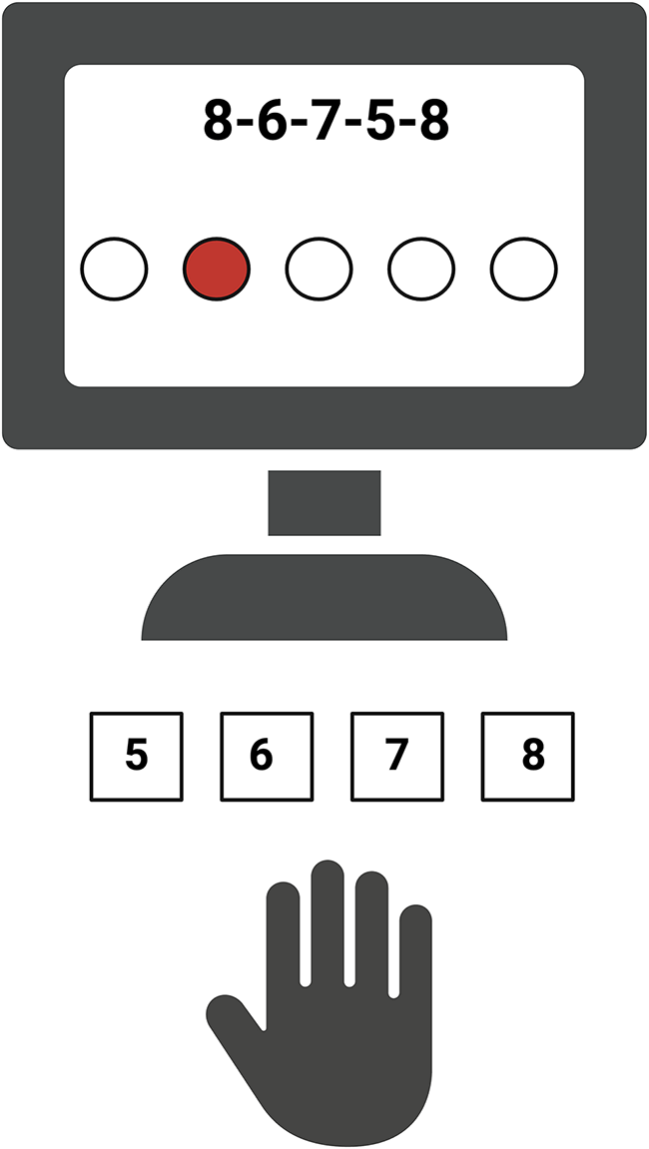
Graphical representation of the finger tapping task. Created with BioRender.com

Motor learning was assessed through a Finger Tapping Task (FTT) [23]. Specifically, FTT is a motor sequence learning task in which subjects are asked to tap with their hand a 5-digit sequence quickly and precisely by using the numerical keys on a computer keyboard. FTT reflects the acquisition of sequential movements progressively structured into a precise and well-articulated behaviour, including a learning and a testing phase. In order to minimize the demand on working memory, the numeric sequence to replicate and five empty circles remain visible at the center of the screen during the entire task. This method offers several advantages: firstly, it standardizes the experimental session by ensuring uniform conditions for all participants [24, 25]; secondly, it provides immediate visual feedback on task progression [24]; and lastly, the view of the sequence on the screen enhances participants’ ability to memorize it [25]. During the task, every time the participant presses a key, one of the circles turns into a red dot regardless of the correctness of the response (i.e., marking only the task progression), to focus on practice-dependent learning and to avoid potential confounders related to different types of motor learning (e.g., reinforcement, error-based) [11, 24]. Figure 1 shows the experimental set-up.

Subjects were seated in a comfortably lit room at a distance of 90 cm from a 20” computer monitor. All participants confirmed they could see both the screen content and the keys to be pressed without difficulty under the proposed lighting conditions. A stimulus presentation was performed by Pyschopy2 [26], and the experimenter monitored the participants’ behaviour throughout the tests. For all subjects, the task was performed with both the affected and unaffected limbs in a different order according to randomization criteria. During the learning phase, participants were required, by explicit request, to memorize a sequence consisting of five numbers (86758). This phase was structured in 6 blocks of 90 s, divided between a resting part, in which a fixation cross simply appeared (30 s), and the motor execution phase, during which the subject was required to perform the sequence (60 s). The learning phase was preceded by a practice phase, in which the participant was asked to complete a test sequence (56785) to clarify how the actual task would be carried out. The participant was asked to be as accurate and fast as possible since measures of accuracy and the number of completed sequences were used to quantify learning performance. Specifically, three parameters of motor performance were derived:

- ACC (accuracy): it expresses the number of sequences correctly completed.
- Seq (sequences): it corresponds to the number of completed five-number sequences, regardless of their correctness.
- ACC/Seq (accuracy/sequences): it combines the two previous parameters and is the percentage ratio between the number of accurate sequences and the number of sequences typed (formula = ACC/Seq*100).

The on-line motor learning ability of the affected and unaffected limb was also investigated for the three variables ACC, Seq, and ACC/Seq by calculating the percentage variation between the average scores of the 5th and 6th block and the 1st block divided by 1st block score. Specifically:

- ACC_on-line= [(ACC V block + ACC VI block)/2 - ACC I block] / ACC I block*100.
- Seq_on-line= [(Seq V block + Seq VI block)/2 - Seq I block] / Seq I block*100.
- ACC/Seq_on-line= [(ACC/Seq V block + ACC/Seq VI block)/2 - ACC/Seq I block] / ACC/Seq I block*100.

While ACC, Seq, and ACC/Seq provided a snapshot of the participant’s performance at specific points in time, the on-line parameters focused on dynamic changes in performance, showing improvement during the task. The on-line learning parameters provide insights of how practice influences motor learning, whereas ACC, Seq, and ACC/Seq provide just a measure of performance at given points.

#### Neuropsychological evaluation, questionnaires and clinical assessment scales

Before the motor task, the patients underwent the following neuropsychological evaluation and questionnaires:

- Stroop Color Word Test (Stroop) - Short Version: it is a simple and quick instrument to assess selective attention, cognitive flexibility, and sensitivity to interference. The Stroop effect is evaluated by calculating a time interference effect and an error interference effect [27];
- Karolinska sleepiness scale (KSS): it assesses the subject’s level of sleepiness during the previous 10 min (in this case, while performing motor tasks). It is based on nine levels, ranging from one, reflecting maximum alertness, and nine, highlighting maximum sleepiness. A score of 7 is considered pathological sleepiness [28];
- Epworth Sleepiness Scale (ESS) is a self-administered questionnaire for assessing the average subjective propensity to sleep during real-life situations. It is considered an indicator of daytime sleepiness [29];
- Pittsburgh Sleep Quality Index (PSQI): the 19 items are grouped into seven composite items that represent the components of the questionnaire, i.e. subjective sleep quality, sleep latency, sleep duration, habitual sleep efficiency, sleep disturbances, use of hypnotic medication, and disturbances during the day. The overall score ranges from 0 to 21, with higher scores indicating more significant sleep quality impairment. The cut-off of sleep disturbance and excessive daytime sleepiness is 5 [30].
- Zung’s Anxiety and Zung’s Depression Self-Assessment Scale: they are aimed at describing the mood during the last week [31]. About anxiety, a total score from 0 to 20 indicates that the level of anxiety could be very low, 21 to 40 low, 41 to 60 moderate, and 61 to 80 high. The Zung’s Anxiety Self-Assessment Scale primarily assesses anxiety state, which refers to how a person feels at a specific moment and can fluctuate in response to various contextual factors. In contrast, anxiety trait represents a more stable aspect of personality and describes a persistent tendency to experience anxiety [32]. Therefore, while anxiety trait reflects a general disposition and has a lesser impact on immediate performance, anxiety state might significantly affect both motor performance and motor learning. Conversely, about depression, scores from 20 to 31 indicate that the level of depression could be very low, 32 to 43 low, 44 to 55 moderate, 56 to 67 high, 68 to 80 very high [31].
- National Institute of Health Stroke Scale (NIHSS): it measures the severity of neurological deficits, with a direct proportionality between score and clinical severity. It analyses level of consciousness, gaze, visual field, facial weakness, limbs motor function, limb ataxia, sensory, aphasia, dysarthria, and extinction [33].

### Statistical analysis

Demographic and psychological characteristics were reported as mean (or frequency) and standard deviation. The variation in the three learning variables across the six performance blocks was analyzed through RM-ANOVA tests with Tukey’s post-hoc correction. Moreover, the interaction of the side (affected or unaffected) and randomization order (unaffected-affected) on ACC, Seq, ACC/Seq, and their online version was revealed with two-way ANOVA tests. Finally, an ANOVA test was performed to assess the possible influence on on-line learning of concordance (i.e. whether the affected upper limb was the dominant one or not), randomization order and interaction between them. The influence of demographic and clinical factors in the learning variables was tested with the Kruskal–Wallis test for categorical variables (gender, lesion type, lesion site, stroke phase) and by correlation tests (Spearman’s rho) for continuous variables. Analyses were conducted with STATA 13, and significance was set at *p* < 0.05.

## Results

### Sample description

#### Demographic and clinical variables

Twenty-six patients were enrolled and evaluated. Table 1 summarises the enrolled patients’ baseline characteristics. Based on previous studies that specifically investigated motor learning in stroke patients, we deemed that this sample size would be adequate to address our research aim [9, 18, 34, 35].

**Table 1:** Baseline characteristics of enrolled patients. * at the time of admission to the Neurology Unit; ^§^ evaluates the correspondence between the manual dominance and the affected limb. *Abbreviations*: NIHSS = National Institute of Health Stroke Scale, SD = standard deviation

	Mean/frequency
Age, years (± SD)	60.15 (10.8)
Gender, no, (male %)	12 (46)
Time since stroke, months (± SD)	39.34 (55.84)
Hemiparesis side (right, no, %; left, no, %)	10 (38.5); 16 (61.5)
Stroke timeframe (subacute, no, %; chronic, no, %)	6 (23.1); 20 (76.9)
Ischemic stroke (%)	23 (88.5)
NIHSS* (± SD)	8.36 (3.53)
Hand dominance (right, no, %; left, no, %)[36]	22 (84.6); 4 (15.4)
Stroke site (cortical, no, %; subcortical, no, %; cortical-subcortical, no, %)	3 (11.6); 12 (46.1); 11 (42.3)
Concordance^§^ (%)	10 (38.5)
Smoking, no (%)	13 (50)
Vascular atheromasia, no (%)	6 (23.1)
Dyslipidaemia, no (%)	9 (34.6)
Hyperhomocysteinemia, no (%)	8 (30.8)
Hypertensive cardiopathy, no (%)	4 (15.4)
Arterial hypertension, no (%)	16 (61.5)

#### Neuropsychological variables (sleep, anxiety, depression, attention)

The mean error and the mean time on the Stroop test were 0.85 (1.22) and 46.47 s (17.39) respectively. The mean score on the KSS was 1.96 (1.11), while on the ESS was 4.30 (2.11). The PSQI score was 11.34 (6.92), indicating a general poor sleep quality and sleep disturbances in this sample [30]. The mean score on the Zung’s Anxiety and Depression Self-Assessment test was 36.92 (7.95) and 38.92 (7.04) respectively, indicative of a low level of anxiety and depressive symptoms [31].

### Motor task

**Fig. 2:**
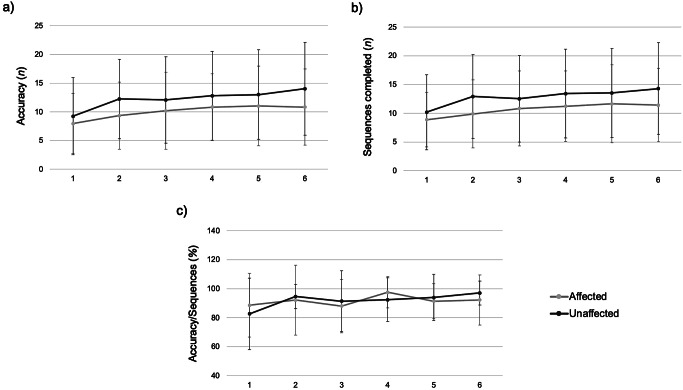
Graphical representation of the average trend of the parameters ACC **(a)**, Seq **(b)**, and ACC/Seq **(c)** at the FTT of the unaffected (dark grey) and affected limb (light grey) in the 6 trials performed. Error bars represents the standard deviation. *Abbreviations*: ACC = accuracy, ACC/Seq = accuracy/sequences, FTT = finger tapping task, Seq = sequences

The FTT was performed with both the affected and unaffected limb: Fig. 2; Table 2 summarise their performance for the ACC, Seq, and ACC/Seq variables. About ACC, a significant block effect (*p* < 0.001, F_5,250_=16.88, η^2^ = 0.252) was shown, whereas no effect was found due to the side (*p* = 0.23, F_1,250_=1.47, η^2^ = 0.00) or the side/block interaction (*p* = 0.33, F_5,250_=1.15, η^2^ = 0.022). There was no difference between the two sides in terms of accuracy. Similarly, about Seq, there was only a significant block effect (*p* < 0.001, F_5,250_=15.72, η^2^ = 0.239). In contrast, the side effect (*p* = 0.23, F_1,250_=1.43, η^2^ = 0.00) and the side and block interaction (*p* = 0.28, F_1,250_=1.25, η^2^ = 0.024) were not significant. Finally, only a significant block effect (*p* = 0.02, F_5,249_ = 2.70, η^2^ 0.051) was evident for the ACC/Seq, in contrast to the side effect (*p* = 0.89, F_1,249_ = 0.02, η^2^ 0.00) and the side and block interaction (*p* = 0.33, F_5,249_=1.14, η^2^ = 0.02). Specifically, about the unaffected limb, there was a progressive and significant increase in accurate sequences (*p* < 0.001), completed sequences (*p* < 0.001), and in their ratio (*p* = 0.01) (see Table 2, Unaffected limb section, and Fig. 2). On the other hand, the affected limb showed a slight increase in accuracy (*p* < 0.001) and in the number of completed sequences (*p* < 0.001), while the ratio ACC/Seq had a reasonably stable trend, except for a peak increase at the fourth block, without significant change (*p* = 0.37) (see Table 2; Fig. 2). Overall, the mean change in accuracy during testing (Acc_on-line) of the affected limb was 41.16% (61.84%) while, for the unaffected one, it was 81.15% (98.11%). About the variation in the number of completed sequences (Seq_on-line), the average was 35.8% (50.55%) in the affected limb, while for the unaffected one, it was 47.28% (44.47%). The final parameter (ACC/Seq_on-line) had a mean change of 1.32% (18.46%) in the affected limb, while for the unaffected one, it had a mean change of 16.54% (35.49%).

**Table 2:** FTT results reported by the unaffected (first section) and by the affected limb (second section). *p < 0.05 Tukey’s post-hoc test compared with the first block. *Abbreviations*: ACC = accuracy, ACC/Seq = accuracy/sequences, FTT = finger tapping task, seq = sequences

Limb	Measure	Block 1	Block 2	Block 3	Block 4	Block 5	Block 6	*P* value
**Unaffected limb**	**ACC**	9.23 (6.73)	12.26 (6.92)*	12.07 (7.54)*	12.8 (7.77)*	13 (7.82)*	14.03 (8.11)*	< 0.001
	**Seq**	10.19 (6.54)	12.92 (7.32)*	12.53 (7.54)*	13.42 (7.73)*	13.53 (7.75)*	14.30 (7.97)*	< 0.001
	**ACC/Seq**	82.59 (24.74)	94.58 (8.4) *	91.35 (20.94)	92.41 (15.18)	93.93 (15.93)	97.01 (8.25)*	0.01
**Affected limb**	**ACC**	7.96 (5.21)	9.34 (5.85)	10.19 (6.74)*	10.84 (5.79)*	11.03 (6.96)*	10.84 (6.67)*	< 0.001
	**Seq**	8.88 (4.73)	9.88 (5.92)	10.84 (6.51)*	11.23 (6.12)*	11.65 (6.78)*	11.42 (6.41)*	< 0.001
	**ACC/Seq**	88.56 (21.99)	92.1 (24.18)	87.98 (18.35)	97.52 (10.86)	91.34 (12.03)	92.22 (17.29)	0.37

### Inter-limb transfer

**Fig. 3:**
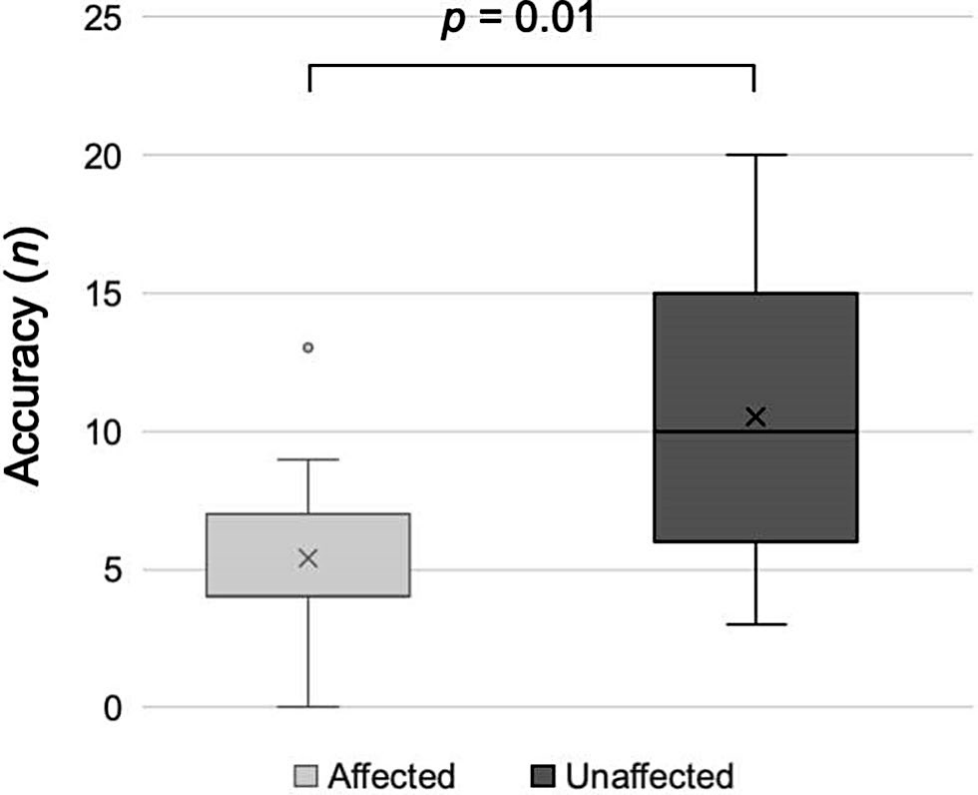
Boxplots of the difference in accuracy at the first block of the FTT between the patients who started the test with the affected (light grey) and unaffected limb (dark grey). Whiskers represent the minimum and maximum values of the sample. The x represents the mean. *Abbreviations*: FTT = Finger tapping task

A trend toward an inter-limb transfer was shown only for the affected side with an interaction of randomization and block (*p* = 0.06, F_5,120_=2.20, η^2^ = 0.08). The analyses showed that patients who started the test with the unaffected limb had a higher accuracy at the first block with the affected one, in particular, 10.53 (5.51) vs. 5.38 (3.42) (*p* = 0.01), as shown in Fig. 3. Finally, the ANOVA showed that concordance didn’t affect the on-line learning (i.e. ACC_on-line) (*p* = 0.217, F_1,22_=1.62, η^2^ = 0.047), whereas randomization (*p* = 0.035, F_1,22_=5.06, η^2^ = 0.147) and its interaction with the concordance (*p* = 0.026, F_1,22_=5.67, η^2^ = 0.165) displayed statistically significant effects.

### Clinical and demographic factors affecting on-line learning

The influence of demographic factors (gender, stroke timeframe, type of stroke, and stroke site) on on-line learning was analyzed. There were no differences between the two sides in the accuracy of the FTT according to gender, stroke timeframe (i.e. subacute or chronic), type of stroke (i.e. ischemic or haemorrhagic), nor according to the stroke site, i.e. cortical, subcortical, or cortico-subcortical. Specifically, all these factors did not influence the performance of the FTT. Similarly, the obtained results showed no correlation between the accuracy in performing the FTT and parameters such as age at the time of the stroke onset, the time elapsed between the event and the assessment, and the NIHSS value calculated at the time of admission for both the affected and unaffected limb. Finally, subjects who made fewer errors on the Stroop test had similar accuracy between the unaffected and affected limbs. In comparison, those who made more errors had higher accuracy in the unaffected limb than in the affected one (rho = 0.35, *p* = 0.09). No difference between the affected and unaffected limb in improving accuracy (ACC_on-line) was recorded in patients who completed the Stroop test without errors. Furthermore, no significant associations were observed between the FTT parameters and ESS, KSS, PSQI, and Zung’s Depression Self-Assessment Scale. A negative correlation was found between the ACC/Seq of the affected side and the score at the Zung’s anxiety Self-Assessment Test (rho=-0.39, *p* = 0.05), showing that subjects with a lower level of anxiety achieved a better trade-off between the accuracy on motor task execution and the number of completed sequences.

## Discussion

One of the most debilitating consequences of stroke is upper limb motor impairment, highlighting the importance of understanding how the CNS learns basic motor strategies post-stroke to optimize rehabilitation strategies. Motor learning is an extremely important field of study in this context, as it makes it possible to characterize how motor performance is acquired and perfected, which is particularly important following stroke [3]. Our results on subacute and chronic stroke patients showed a slight but stable increase in the accuracy and number of completed sequences in a FTT performed with the affected limb. As expected, both the unaffected and affected limb improved in these parameters, confirming that the performance enhancement depends on the amount of practice [37]. Our results are particularly relevant, suggesting that learning sequence-specific motor skills might be an important stimulus to remedy maladaptive patterns of brain activity after stroke [38]. Moreover, our results highlight the potential for motor recovery even in stroke patients in the chronic phase. Previous studies in chronic stroke patients showed that Constraint-Induced Movement Therapy improves motor performance through exercises that offer a proportionally increasing ‘challenge’ to the paretic limb movement [39]. Our data emphasize the ability of stroke patients to retain motor skills even long after the acute event; furthermore, a simple tool as the FTT seems able to improve motor learning through repetition of sequences, without any additional challenges. Indeed, although various tasks are currently available for assessing motor learning abilities in stroke patients, the FTT offers several advantages. It is easy to understand and execute, without requiring high cognitive load [23]. Additionally, it allows the assessment of fine motor control and finger movements, frequently impaired in stroke patients [40, 41]. FTT provides a standardized non-operator dependent evaluation easily implementable in clinical practice. Indeed, it only requires a computer support and a user-friendly software for the collection of relevant measurements, representing a promising tool for the assessment of motor learning in the neurorehabilitation field.

Improvements in motor performance are reasonably due to use-dependent learning. This simple, goal-independent process forms motor memories to make movements more similar to the previous one [11]. Our results show that this type of motor learning is maintained also in stroke patients, even with a simple task and a small number of repetitions. Considering that on-line practice-dependent learning represents a prerequisite for the subsequent retention and transfer phases, our study confirms that even patients far from the true recovery phase (i.e. the acute timeframe characterized by augmented neuroplasticity) can improve their motor skills enhancing their daily performance [11].

**Fig. 4:**
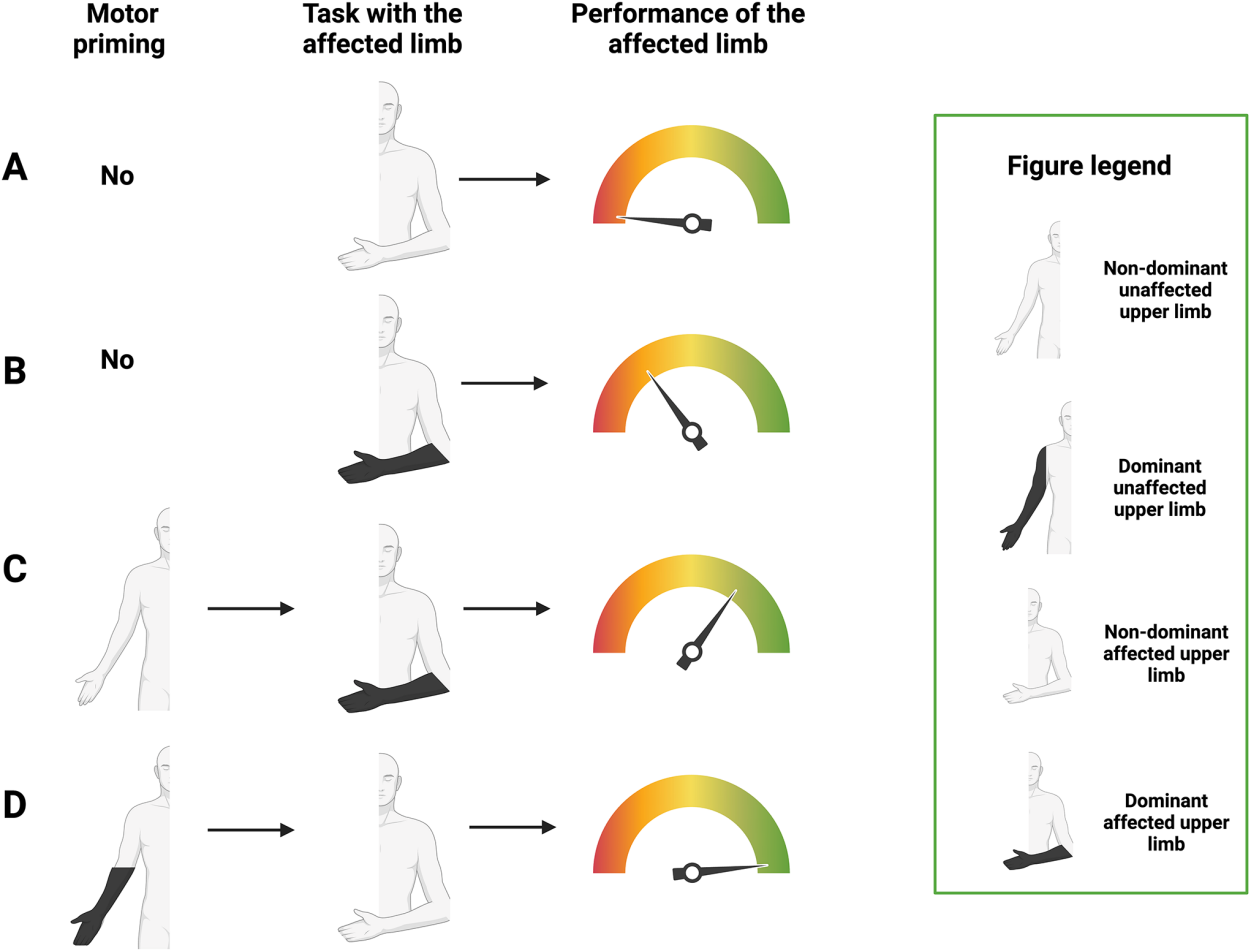
Graphical representation of the hypothesised model of inter-limb transfer in stroke patients. The first column depicts the effect of randomization (i.e., performing the task first with the affected or unaffected limb) and concordance (i.e., whether the dominant hand matches the paretic one) on the performance of the affected upper limb (second column displaying ACC_online). A and B show conditions where the task is performed first with the affected upper limb, resulting in low or moderately low performance depending on whether the paretic hand does not match **(A)** or matches **(B)** the dominant one, respectively. Conversely, C and D show conditions where the task is performed first with the unaffected limb, assessing the effects of this motor priming on subsequent performance of the affected limb. Considering the hypothesized inter-limb transfer of learning, the performance of the affected upper limb is moderately high if the unaffected limb does not match the dominant one **(C)** and high if it matches the dominant one **(D)**. C and D suggest that better performance with the first limb (the unaffected and dominant limb) corresponds to greater motor pattern consolidation and performance of the affected limb. Created with BioRender.com

With our study we highlighted an interesting difference between the affected and the unaffected limb in the ratio accuracy/completed sequences. Specifically, the affected limb didn’t increase in accuracy respect to the number of completed sequences; conversely, the unaffected limb increased its accuracy more markedly than the relatively less pronounced increase in the number of completed sequences. We can hypothesize that this result reflects a resource allocation strategy. Specifically, the affected limb, with lower maximum resource compared to the unaffected one, has to compromise between the accuracy and the number of completed sequences; instead, the unaffected one, which can complete a fair number of sequences, can invest more in accuracy. Finally, we can assume a crucial role of attention in this process. The subjects who completed the Stroop test with few errors showed similar accuracy between the two limbs; those who made more errors showed higher accuracy of the unaffected limb than the affected one. Given the pivotal role of attention in motor performance, it’s unsurprising that deficits in attention have a more pronounced impact on the affected limb’s performance compared to the unaffected one. The latter typically maintains accuracy owing to the preservation of motor patterns, requiring less cognitive load from the patient [12, 42]. Furthermore, when subjects started the FTT with their unaffected limb, they showed higher accuracy in the subsequent task performed with the affected limb compared to subjects who started with the affected one, reaching values near to the statistical significance (*p* = 0.06). This finding shows that, when the motor task is initially performed by the unaffected limb, it better fixes the model to be learned and transfer to the affected one, resulting in higher accuracy compared to when the affected limb performs the task first. The inter-limb transfer is a phenomenon in which a training effect is transferred from a trained limb to an untrained one [43] but its rehabilitative implications have only been partially explored. Indeed, few studies have investigated the effect of inter-limb transfer to improve dexterity performance of the paretic upper limb in post-stroke patients [19, 44]. In the clinical setting, training of the impaired limb has been considered an essential component of rehabilitation, based on evidence supporting the importance of use-dependent and repetitive training effects [45, 46]. However, the inter-limb effect could represent an excellent therapeutic strategy in patients with severe limb paralysis, making the interest in its application recently growing [47]. Notably, we also highlighted that, while hand concordance (whether the affected hand matches the dominant one) doesn’t affect on-line performance (i.e., ACC_online), it significantly interacts with randomization order. This suggests that the motor pattern is most effectively executed (and consequently best transferred to the contralateral side) when initially performed with the unaffected dominant hand. These data support the usefulness of this rehabilitation strategy after the acute stroke timeframe, with interesting consequences from a functional point of view. For example, when a stroke affects the right brain hemisphere in a patient with right-hand dominance (i.e., the most common in the general population), this model suggests that starting the rehabilitation treatment with the dominant (i.e., unaffected) limb in this situation will yield the maximum clinical benefit for the subsequent performance of the affected limb. Figure 4 summarises the hypothesized model of inter-limb transfer based on our results.

About sleep, anxiety, and depression, no relevant correlations were found with the FTT performance, probably due to the complexity and multifactorial nature of these aspects, only partially investigated by questionnaires [48]. Additionally, the patients recruited exhibited satisfactory sleep quality and mild levels of depression based on questionnaire scores. Only for the affected limb we highlighted a correlation between ACC/Seq and anxiety, particularly subjects with a lower level of anxiety achieved a better compromise between the accuracy of motor task execution and the number of completed sequences. While both anxiety state and trait might be relevant in this context, the first seems to be more relevant for understanding its impact on motor learning performance in stroke patients. In fact, a recent study proved that stroke patients with high level of anxiety state were more adversely affected by challenging dual-task conditions [49]. This focus allows for more targeted and effective intervention strategies during rehabilitation and aligns with existing literature suggesting that tasks demanding rapid performance can elevate anxiety levels in subjects, potentially resulting in performance decline [50]. Individuals experiencing high levels of anxiety are unable to offset the cognitive deficit induced by their concern, resulting in diminished benefits from practice and reduced learning outcomes. Furthermore, there might exist a bidirectional causality between anxiety and performance, as worries could stem from perceived inadequate performance, subsequently impacting performance further by disrupting attentional focus [51]. Finally, it is conceivable that, among high-anxiety individuals, some exhibit sufficient competencies but show poor performance due to anxiety, while others may exhibit poor ineffective practice strategies, contributing to their learning or performance deficits and generating anxiety [52].

The present study has some limitations that may affect the interpretation of our results. Firstly, we recorded data from a small sample not homogeneous for several aspects (time since stroke and performance level). However, we merged findings from both stroke patients groups (i.e. subacute and chronic) since evidence suggest that neuroplasticity is most pronounced in the acute and early subacute stroke phases, whereas our subacute patients were primarily between two and three months post-stroke, nearing the conclusion of this interval [53] Consistently, subgroup analysis comparing subacute and chronic patients did not reveal statistically significant differences, reinforcing this choice. Secondly, we recruited only subjects with mild to moderate hemiparesis due to motor competencies required for the execution of the FTT and excluding a broad spectrum of stroke survivor with poor motor skills. Thirdly, we performed a single assessment, whereas motor learning paradigms commonly encompass three discrete phases: an acquisition session involving motor skill practice, a maintenance session evaluating skill performance following rest intervals, and a transfer test to assess skill generalization to similar or varied tasks [7]. However, literature supports our choice of a single assessment as adequate for characterizing motor learning in stroke patients [9]. Furthermore, as our study only assessed motor performance and the potential for improvement through practice-dependent learning in the context of a specific task (i.e., the FTT), it cannot provide data on the generalizability of these improvements to other tasks (e.g., the ability to increase autonomy in ADLs). However, it seems reasonable to hypothesize that these patients could leverage these abilities to enhance their performance in various daily activities in which manual dexterity is often crucial [54]. Further studies are needed to evaluate the correlation between improvements in FTT performance and autonomy in daily life. In addition, motor learning is an extremely complex phenomenon affected by numerous factors and variables, which are difficult to control entirely in the clinical setting [55]. It is also important to note that the discussed inter-limb transfer effect did not reach statistical significance, although the observed trend approached it closely. Likely, the aforementioned limitations prevented us from achieving significance. Therefore, these implications should be interpreted with caution, and further research will be required to confirm these findings and develop robust models of their clinical practice applications. Finally, a significant number of the recruited patients were inexperienced with computer use, increasing the complexity of the evaluation and exacerbating difficulties and anxiety, with potential negative impact on performance [56].

## Conclusions

Our findings highlighted that, in stroke patients, motor learning of the upper limb is maintained even in the subacute and chronic phases but is reduced in the affected one. Notably, the preservation of the on-line practice-dependent learning suggests that even patients far from the true recovery phase can acquire effective compensation strategies to improve their motor performance. Among the factors potentially affecting motor learning, only attention and anxiety were confirmed as influencing learning, probably due to their complex and multifactorial nature difficult to capture entirely with simple questionnaires. The inter-limb transfer phenomenon could be a useful strategy for the rehabilitation of stroke patients, even in the chronic timeframe. Considering future applications, it would be interesting to carry out an analysis of neural correlates of FTT, in order to observe the brain circuits involved in its modifications during learning. Finally, it might be useful to validate this motor task as an outcome measure in stroke patients, both in research and clinical settings.

## Data Availability

The data that support the findings of this study are available from the corresponding author upon reasonable request.
